# Alchemical free energy simulations without speed limits. A generic framework to calculate free energy differences independent of the underlying molecular dynamics program

**DOI:** 10.1002/jcc.26877

**Published:** 2022-04-29

**Authors:** Marcus Wieder, Markus Fleck, Benedict Braunsfeld, Stefan Boresch

**Affiliations:** ^1^ Department of Pharmaceutical Sciences, Faculty of Life Sciences University of Vienna Vienna Austria; ^2^ Department of Computational Biological Chemistry, Faculty of Chemistry University of Vienna Vienna Austria

**Keywords:** free energy calculations, solvation free energy, toolkit

## Abstract

We describe the theory of the so‐called common‐core/serial‐atom‐insertion (CC/SAI) approach to compute alchemical free energy differences and its practical implementation in a Python package called Transformato. CC/SAI is not tied to a specific biomolecular simulation program and does not rely on special purpose code for alchemical transformations. To calculate the alchemical free energy difference between several small molecules, the physical end‐states are mutated into a suitable common core. Since this only requires turning off interactions, the setup of intermediate states is straightforward to automate. Transformato currently supports CHARMM and OpenMM as back ends to carry out the necessary molecular dynamics simulations, as well as post‐processing calculations. We validate the method by computing a series of relative solvation free energy differences.

## Graphical abstract

We describe the theory of the common‐core/serial‐atom‐insertion (CC/SAI) approach to compute alchemical free energy differences and its practical implementation in a Python package called Transformato. CC/SAI is not tied to a specific biomolecular simulation program and does not rely on special purpose code for alchemical transformations. Transformato currently supports CHARMM and OpenMM as back ends to carry out the necessary molecular dynamics simulations, as well as post‐processing calculations.

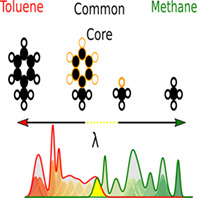

## 1 INTRODUCTION

The free energy difference between two states determines their relative stability; applied to a (bio)chemical reaction it determines the direction in which the reaction will take place voluntarily. While the above ignores complications from kinetic effects, such as reaction barriers, the capability to compute free energy difference between reactants and products permits the prediction of equilibria of chemically and biochemically relevant processes. Great efforts have been and are being exerted to compute free energy differences of, for example, binding, solvation, and partitioning reliably and reproducibly. The tool of choice to compute these quantities are so‐called free energy simulations (FES), which are rapidly becoming a standard tool in computational chemistry.^1^, ^2^, ^3^, ^4^, ^5^

The ever‐increasing speed of hardware, in particular the raw computational power of consumer graphics cards, combined with algorithmic progress today make it possible to carry out molecular dynamics (MD) simulations of biomolecular systems, which 15 years ago would have been possible only on the world's most powerful computers. This development has increased the usage of MD simulations as a standard tool even by non‐experts.^6^ FES, even though in most cases MD based, have until recently not profited to the same degree from the increase in computational speed. First, FES incur some principal overhead; for example, in many programs the reciprocal space energies and forces of the (particle‐mesh) Ewald sum need to be computed twice, once for the initial, once for the final state at each step of a FES. Further, to compute free energy differences between states, various tricks are needed which require specialized computer code/routines. These capabilities were or are often not available in the fastest code paths of many widely used MD programs. Therefore, MD simulations used to compute (alchemical) free energy differences often are slower than “plain” simulations of the same system. An overview concerning the computational overhead of FES can be found in Ref. 7 Clearly, it would be highly desirable to run the sampling phase of FES at a similar speed as regular MD simulations. Progress is being made and GPU support for alchemical FES is becoming available.^4^, ^8^

Another obstacle to the more widespread use of FES is setting up the transformation between two states, that is, how to change state A into state B. Tools have been developed to aid with this step; an early example is the (now defunct) FESetup web server,^9^ which handled the details to set up alchemical transformations for several simulation programs, such as AMBER,^10^ CHARMM,^11^ GROMACS,^12^ or NAMD.^13^ A principal problem, however, remains: each program that supports alchemical FES has its own internal approach to how transformations are set up; for example, is the transformation accomplished by a single vs. dual topology approach,^14^ or—in case of single topology—is the mixing done on the level of parameters or energies/forces.^7^ Each of these approaches has different strengths and weaknesses. Thus, a particular transformation may be easy to set up in one program, but difficult to accomplish in another program. In other cases, the opposite may be true. The practical difficulties resulting from this are illustrated in two very recent publications.^15^, ^16^ Loeffler et al. compared results for several relative free energy differences of solvation computed with AMBER, CHARMM, GROMACS, and SOMD.^17^ While the final, overall agreement was good, the authors stressed that considerable effort was needed to achieve it, and they pointed out several “quirks” of each of the programs considered. Rizzi et al.^16^ focused on convergence and reproducibility of binding free energy methodology of multiple programs starting from a single set of parameter files, partial charges, and initial geometries of host‐guest systems in the course of the SAMPL6 SAMPLing challenge. They observed differences between converged binding free energy estimates ranging from 0.3 to 1.0 kcal/mol, highlighting the challenges that the field faces when it comes to the transferability of results between different free energy codes.

In this study, we show how the issues just outlined (computational overhead, reproducibility, and a sometimes inflexible corset to set up alchemical transformations) can be circumvented by avoiding dedicated codes to compute free energy differences. When the first GPU accelerated MD codes became available, Boresch and Bruckner presented and tested such an approach, which enabled them to compute alchemical free energy differences using programs without the functionality for this task.^18^ The approach was limited to the calculation of absolute solvation free energy differences, and the necessary steps to set up such calculations included manual modifications of parameter and topology files, requiring expert knowledge both of free energy calculations and the inner workings of CHARMM. Despite that, we have continued to use it on occasion^19^; a recent study by Giese and York^7^ utilized related ideas. Here we extend the approach of Ref. 18 to the case of, in principle, arbitrary alchemical transformations. We have developed a Python package (Transformato) to set up the intermediate steps leading from a state A to B. The tool generates force field parameters and systems information as needed (for how to obtain the code and data we point to the data availability statement). The underlying MD program is not carrying out any “alchemical FES” specific tasks; that is, it is carrying out a straightforward MD. All quantities needed to compute the free energy differences of interest are obtained using Transformato in post‐processing steps from trajectories saved during the MD simulations.

In order to facilitate the task of setting up intermediate states, we adopt the following approach to the computation of a free energy difference between two states A and B. Rather than alchemically transmuting A into B, we search for a suitable common substructure, which is (mostly) identical in the two systems (molecules). We refer to this as the common core (CC); we stress that CC of A (${\mathrm{CC}}_{A}$) and B (${\mathrm{CC}}_{B}$) need not be described by identical force field parameters as long as there is correspondence between the atoms of the CCs (see Section 2 for details). Assuming for the moment the simplest case, that is, ${\mathrm{CC}}_{A}\equiv {\mathrm{CC}}_{B}≐\mathrm{CC}$, this means that the free energy difference Δ*G*(*A* → *B*) is carried out in two steps, $\Delta G\left(A\to \mathrm{CC}\right)+\Delta G\left(\mathrm{CC}\to B\right)$, where the second step is in practice computed as $-\Delta G\left(B\to \mathrm{CC}\right)$. The use of a CC facilitates the setup of the alchemical transformation considerably because this allows us to define the physical end‐states without dummy atoms and dummy parameters. Additionally, it is a good match for the serial atom insertion (SAI) approach of Boresch and Bruckner,^18^ which is employed here as well. Further, if free energy differences between more than two states are needed, for example, solutes or ligands *L*_1_, *L*_2_, *L*_3_,…*L*_n_ and provided a suitable CC exists, then one needs exactly *n* FES to compute all possible relative free energy differences between the *n* states.

We test the approach by recomputing all relative solvation free energy differences reported in Ref. 15 To highlight the generality of the approach, we report results carried out with CHARMM and OpenMM as the underlying MD program. The use of OpenMM, a program, or rather a library for MD simulations, illustrates the versatility since the base OpenMM suite has practically no provisions for alchemical FES, which is shipped separately in the OpenMMTools^1^ or Perses package^2^.

The remainder of the manuscript is organized as follows. In Section 2, we first provide full details of the CC approach. In particular, we demonstrate that contributions from so‐called dummy atoms, which typically are present in ${\mathrm{CC}}_{A}$ and ${\mathrm{CC}}_{B}$, and which constitute one difference between the two “common” cores, cancel from parallel legs of the thermodynamic cycles usually employed in applications of alchemical FES. Section 2 concludes with a review of SAI. While the CC concept is crucial to our approach, we use SAI out of practical necessity—SAI could be replaced by soft‐core potentials if these are available without impeding computational speed. In Section 3, we first present the benchmark systems of Ref. 15, followed by a detailed description of the simulation details. The presentation of Results (Section 4) is followed by a Concluding Discussion (Section 5).

## 2 THEORY

**FIGURE 1:**
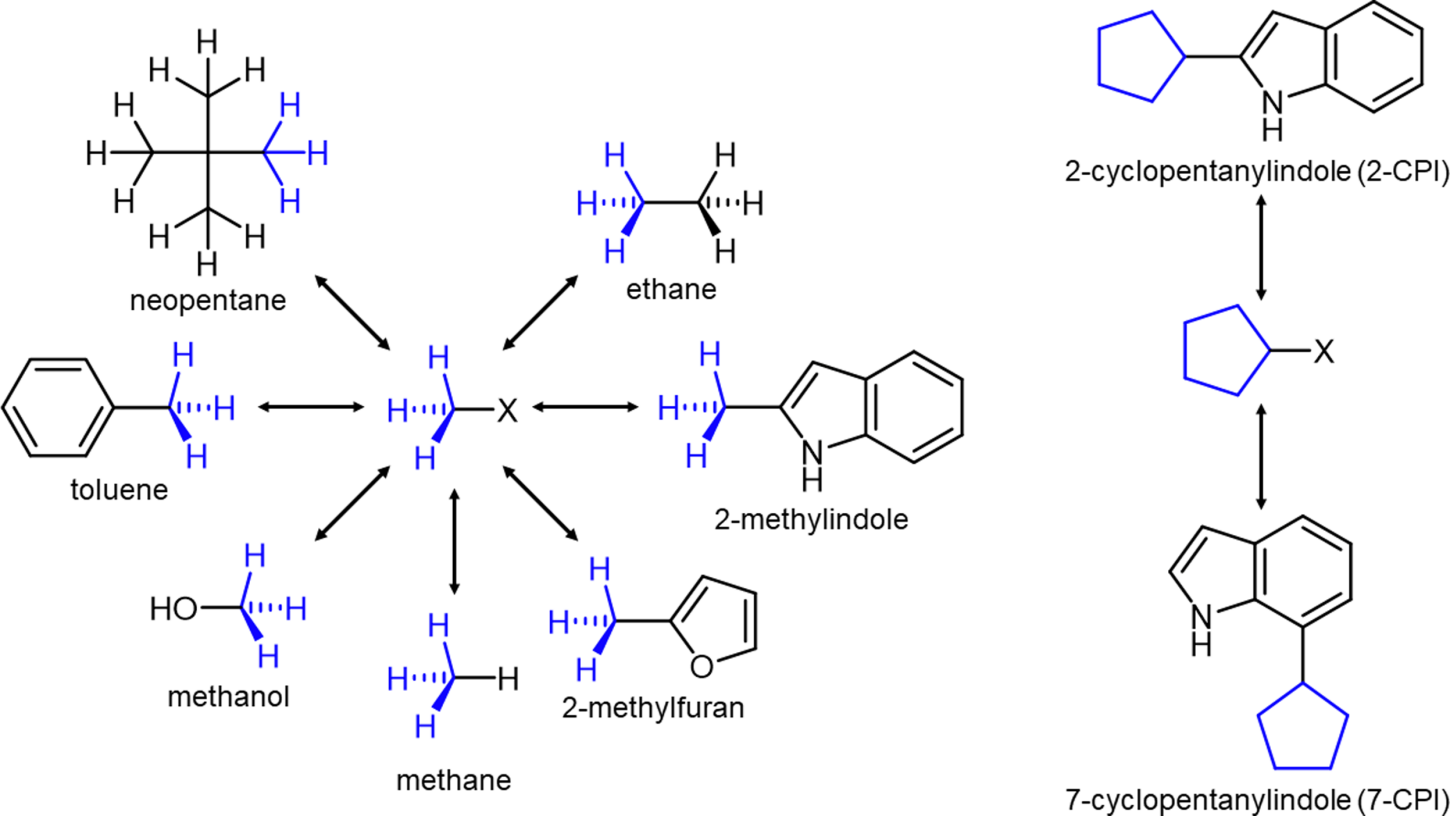
Model systems used in relative solvation free energy calculations. Two different common cores were used, a methane‐like molecule (${\mathrm{CH}}_{3}X$) for the seven solutes shown on the left, and a modified cyclopentane for the transformation of 2‐cyclopentylindole (2‐CPI) to 7‐cyclopentylindole (7‐CPI).

*Common cores are used to connect the physical end‐states of different molecular typologies*. The central and technically challenging step of a FES is setting up the alchemical transformation between two or more molecules or chemical moieties, for example, the transformation of ethane to methanol. Often, if free energy differences for a series of solutes or ligands need to be computed, there is some common substructure. For example, in one of our model applications, shown on the left of Figure 1, a methyl (CH${}_{3}$–) moiety is present in all compounds. Let us denote this situation as CC‐R${}_{i},\;i=1,\ldots N$, where CC indicates the common substructure, and R${}_{i}$ the parts in which the molecules differ. To compute the free energy differences between *N* molecules, one has to carry out at least *N* ‐ 1 alchemical transformations,

(1) $$\mathrm{CC}‐R_{1}\to \mathrm{CC}‐R_{i}\;\left(i=2,\ldots ,N\right).$$

If for example, the CC‐R${}_{i}$ corresponds to ethane, neopentane, toluene, and so forth. from our example, then each of the transformations in Equation 1 must be set up individually. This is prone to error if done by hand and challenging to automate. Of course, in such a situation practitioners will choose the alchemical paths/transformations that are easiest to set up; for example, in the study which inspired this model application, Loeffler et al. mutated each of the larger molecules to methane, the smallest compound of the set.

The strategy to always mutate towards the smallest common denominator, that is, going to the smallest compound containing the common substructure (CH${}_{3}$– in our example) can be generalized and forms the basis of what we refer to as the CC approach. Rather than setting up a transformation $\mathrm{CC}‐R_{A}\to \mathrm{CC}‐R_{B}$ in a single step, we break it up into two mutations:

$$\mathrm{CC}‐R_{A}\to \mathrm{CC}‐{\mathrm{DR}}_{A}\;\left(\Delta G_{A}\right)$$

(2) $$\mathrm{CC}‐R_{B}\to \mathrm{CC}‐{\mathrm{DR}}_{B}\;\left(\Delta G_{B}\right)$$

In each of the steps, the functional group R${}_{i}$ is mutated to non‐interacting atoms DR${}_{i}$, commonly referred to as dummy atoms. Provided they are treated correctly, dummy atoms have no influence on the result of double free energy differences, as calculated in the usual thermodynamic cycles.^20^ While the partition function and, hence, the free energy of “CC” and “CC‐DR${}_{i}$” are different, the dummy atoms give an additive contribution to the partition function, which cancels from the double free energy differences of interest. From this, it follows that any double free energy difference $\mathrm{\Delta \Delta G}\left(\mathrm{CC}‐R_{A}\to \mathrm{CC}‐R_{B}\right)$ can be split into the two steps of Equation 2. Both have to be computed for each leg of the thermodynamic cycle of interest. Specifically,

(3) $$\Delta \Delta G\left(\mathrm{CC}‐R_{A}\to \mathrm{CC}‐R_{B}\right)=\Delta \Delta G\left(\mathrm{CC}‐R_{B}\to \mathrm{CC}‐{\mathrm{DR}}_{B}\right)-\Delta \Delta G\left(\mathrm{CC}‐R_{A}\to \mathrm{CC}‐{\mathrm{DR}}_{A}\right)$$

We stress that Equation 3 holds even when the number of dummy atoms is different for A and B.

From a practical point of view, each of the alchemical transformations arising in the CC approach consists of mutating one or more atoms to dummy atoms. In contrast to a general alchemical transformation, this is straightforward to set up and, most importantly, easy to automate.

There are cases in which Equation 3 is not sufficient. As mentioned in the Introduction, the CC needs not to be exactly the same in a series of transformations $\mathrm{CC}‐R_{i}\to \mathrm{CC}‐{\mathrm{DR}}_{i}$. Consider the following scenario: element identity (i.e., two nodes of the molecular graph match if they have the same element regardless of atom type or hybridization) is used as the matching criterion for the CC. When starting with several physical molecules $\mathrm{CC}‐R_{i}$, one or more atoms of the CC may be represented by different atom types of the force field, and/or their partial charges may be different. In other words, having transformed the $‐R_{i}$ to the respective dummy groups $‐{\mathrm{DR}}_{i}$, there may be small differences in the CCs, that is, the endpoints must be written as ${\mathrm{CC}}_{i}‐R_{i}$. These ${\mathrm{CC}}_{i}$, however, must have the same number of atoms, as well as a one‐to‐one correspondence between each of the atoms. Permitting such flexibility makes it easier to find/define CCs.

Assuming that we have two such endpoints, ${\mathrm{CC}}_{A}$ and ${\mathrm{CC}}_{B}$, then obviously the free energy difference between them must be accounted for. In principle, this can be done in a separate step/calculation by computing $\Delta \Delta G\left({\mathrm{CC}}_{A}\to {\mathrm{CC}}_{B}\right)$ and adding it to Equation (3). Alternatively, one can add additional alchemical mutation steps after each transformation to ${\mathrm{CC}}_{i}$, coercing them into a single CC. While the ${\mathrm{CC}}_{i}$ need not be identical, they are likely very similar, so the additional transformations do not require many steps. This is the procedure we used in all examples considered in this study; that is, our transformations always follow the pattern ${\mathrm{CC}}_{i}‐R_{i}\to {\mathrm{CC}}_{i}‐{\mathrm{DR}}_{i}\to \mathrm{CC}‐{\mathrm{DR}}_{i}$ for i=A,B.

If we applied this approach naively to our model application of Figure 1, we would choose the CH${}_{3}$– moiety as the CC. While theoretically correct, this results in technical difficulties maintaining the dummy group DR in a meaningful position and orientation relative to the CC. In our recent analysis,^20^ we classified a dummy atom configuration as in CH${}_{3}$‐DR as a “triple junction.” This is the one case where the required decoupling between degrees of freedom of the dummy atom and of the physical atom is difficult to accomplish. By contrast, the easiest to handle case in this respect is the “terminal junction.” The triple junction configuration can be avoided by maintaining one atom of the ‐DR${}_{i}$ group as interacting. The “better” CC, therefore, is CH${}_{3}$X‐ as shown in Figure 1. Specifically, the alchemical transformation to mutate, for example, ethane into this CC becomes ${\mathrm{CH}}_{3}{\mathrm{CH}}_{3}\to {\mathrm{CH}}_{3}{\mathrm{XD}}_{3}$. In our example, methane (“CH${}_{3}$H”) would be a valid CC (with the fourth hydrogen being the X), but the ability to choose and adapt the interaction parameters of X offers additional flexibility. For the specific parameters used for X in this work, see Section 3.

“*Serial atom insertion*” *is used to avoid the need for customized soft core potentials*. When creating or annihilating particles in a dense environment, such as solvent, the van der Waals endpoint problem occurs.^21^ In FES the standard workaround is the introduction of a soft‐core potential.^22^, ^23^, ^24^ The corresponding computer code, however, is often interlaced with the FES code, so, as described in the introduction, GPU support may be poor or missing. Therefore, in the present work, we rely on the so‐called SAI method to avoid van der Waals endpoint problems.^18^ Instead of scaling Lennard–Jones (LJ) interactions as a function of a continuous coupling parameter, the LJ interactions of an atom (the partial charge of which was switched to zero in a previous step) are either fully interacting or completely turned off. Using Bennett's acceptance ratio method (BAR)^25^ or its multi‐state extension MBAR,^26^ the free energy difference between one or even two LJ interactions being turned on/off can be computed reliably. As described in Ref. 18, SAI is incompatible with thermodynamic integration since unmodified LJ potentials are used and the intermediate states are no longer continuous with respect to the coupling parameter *λ*. It should be stressed that SAI is not essential to the CC approach; however, since it does not require specialized code as the soft‐core potential, the combination of CC and SAI makes it possible, in principle, to set up FES on top of any biomolecular MD program.

## 3 METHODS

### 3.1 Overview of calculations

Our model/benchmark calculations involve the same nine molecules used by Loeffler et al.^15^ Rather than computing specific relative solvation free energy differences (between ethane, methanol, neopentane, toluene, 2‐methylfurane and 2‐methylindole relative to methane, and between 2‐cyclopentanylindole (2‐CPI) and 7‐cyclopentanylindole (7‐CPI) directly as in Loeffler et al.), we inserted a methane‐like and a cyclopentane‐like CC as shown in Figure 1. All relative FES in the CC/SAI framework were carried out with CHARMM^11^ using the domain decomposition implementation for GPUs^3^ and OpenMM.^27^ Calculations were repeated five times and the average and standard deviation of the individual MBAR free energy estimates are reported.

We re‐parameterized the solutes using the CGenFF interface at paramchem.org ^28^, ^29^, ^30^; therefore, the relative solvation free energy differences calculated in this work cannot be directly compared to the results of Loeffler et al.^15^ To validate the workflow with an established protocol, we computed the absolute solvation free energies for all nine solutes with the PERT module of CHARMM.^11^ The use of PERT introduced a subtle complication because in this module only the original CHARMM switching function for LJ interactions (from now on referred to as “vswitch”)^31^ but not the LJ force switching function (“vfswitch”)^32^ is supported. OpenMM, on the other hand, neither has native support for CHARMM's “vswitch” nor for “vfswitch.” However, when obtaining input scripts for OpenMM through the CHARMM‐GUI server,^33^, ^34^ a custom energy routine for “vfswitch” is provided. In fact, many of our inputs both for CHARMM and OpenMM are based on scripts generated by CHARMM‐GUI to maintain as much consistency as possible between the two programs. Our testing/validation, therefore, proceeded as follows. First, we compared relative solvation free energy differences obtained in the CC/SAI framework with CHARMM, truncating LJ interactions with “vswitch”, and compared these results to the corresponding differences between absolute solvation free energies obtained with PERT. Then, we compared relative solvation free energies calculated with the CC/SAI framework using CHARMM and OpenMM directly with each other, truncating LJ interactions with “vfswitch.”

### 3.2 Relative alchemical free energy calculations using Transformato

We have developed a Python package named Transformato that performs the steps required by the CC/SAI approach. Transformato takes care of generating the alchemical path, dispatches sampling and post‐processing calculations at the alchemical states, and computes the relative free energy difference between physical states and their CC. At present, Transformato can perform these tasks mostly automatically using either OpenMM or CHARMM for sampling and post‐processing.

To calculate the free energy from two physical end‐states, the following steps have to be performed:

1. Identify the maximum common substructure using a specific node/vertex matching criteria;
2. Generate the alchemical path connecting the molecules to the same CC;
3. Sample the alchemical states;
4. Use the multi‐state Bennett acceptance ratio estimator implementation in pymbar^26^ to calculate the free energy difference from the alchemical samples obtained in step (3).

#### 3.2.1 Identifying the maximum common substructure

Transformato identifies the CC substructure using a customized maximum common substructure algorithm based on the chemoinformatics toolkit RDkit,^35^ with added checks to avoid ring breaking and enabling user‐defined and customized atom matching criteria. In the calculations presented here, a methane‐like and a cyclopentane‐like CC were used. These consist of a methyl or cyclopentanyl group, respectively, to which a junction LJ particle, denoted as X, is connected, as shown in Figure 1. We computed relative solvation free energies between methane, methanol, ethane, 2‐methylfuran, 2‐methylindole, neopentane, and toluene to the methane‐like CC and for 2‐cyclopentanylindole (2‐CPI) and 7‐cyclopentanylindole (7‐CPI) to the cyclopentane‐like CC. As described in Section 2, the junction LJ particle serves as the last non‐interacting atom connecting the dummy atom region of the molecule with the real region. In both cases, its presence changes a “triple” into a “terminal junction,” guaranteeing that the dummy atoms present in the CC states have no influence on the result.^20^ Its LJ parameters were set to *ϵ* = −0.15 kcal/mol and *σ* = 1.5 *Å*, and its partial charge was zero. The bonded parameters involving X and the CC atoms were those of the corresponding hydrogen in methane and cyclopentane, respectively.

#### 3.2.2 Defining the alchemical path

To generate the alchemical path connecting the physical end‐state of two molecules to their CC, at least for one molecule a non‐zero number of atoms have to be transformed into dummy atoms. Optionally, CC parameters have to be modified so that the end‐state is the same for both transmutations. Transformato always changes a molecule or chemical moiety from the initial physical state to the target CC in the following order. First, we turn off the Coulomb interactions of all atoms not in the CC, including the junction atom X. This step corresponds to linearly scaling a traditional coupling parameter *λ* from 1 to 0, but Transformato writes explicit charge information (into the PSF file read by both CHARMM and OpenMM for every intermediate step). If the charge manipulation results in a non‐integer net charge, a compensating charge of opposite sign is applied to the physical atom (not the junction atom X) that connects the dummy region with the interacting region.

LJ interactions of the atoms not part of the CC are turned off by SAI.^18^ In the systems studied here, we scaled the van‐der‐Waals interactions of all hydrogen to zero in a single step. Next, one or at most two of the remaining heavy atoms were turned off per step. Proceeding in this manner resulted in sufficient overlap with neighboring states.

For the systems studied here, turning off Coulomb and LJ interactions of all atoms not in the CC and if necessary transforming the junction atom X to a LJ particle (cf. above) resulted in CCs which only differed in the bonded parameters involving X. The bonded parameters involving X were those of the original atom. Choosing “methane” (CH${}_{3}$X) and “cyclopentane‐X" (cf. above) as the final end‐states (target CCs) as described earlier, one has to change the bonded parameters involving X of all other endpoints; this corresponds to transforming CC_*i*_ to CC. In each case, the force field parameters of the bonded terms involved were scaled linearly. In more complex scenarios additional modifications might be necessary to transform the intermediate state CC_*i*_ reached after turning off charges and LJ interactions of non CC‐atoms into the target CC. For each of the intermediate states parameter and topology information was written out in CHARMM format (PSF/PRM/RTF files) using the ParmEd library.^36^

**FIGURE 2:**
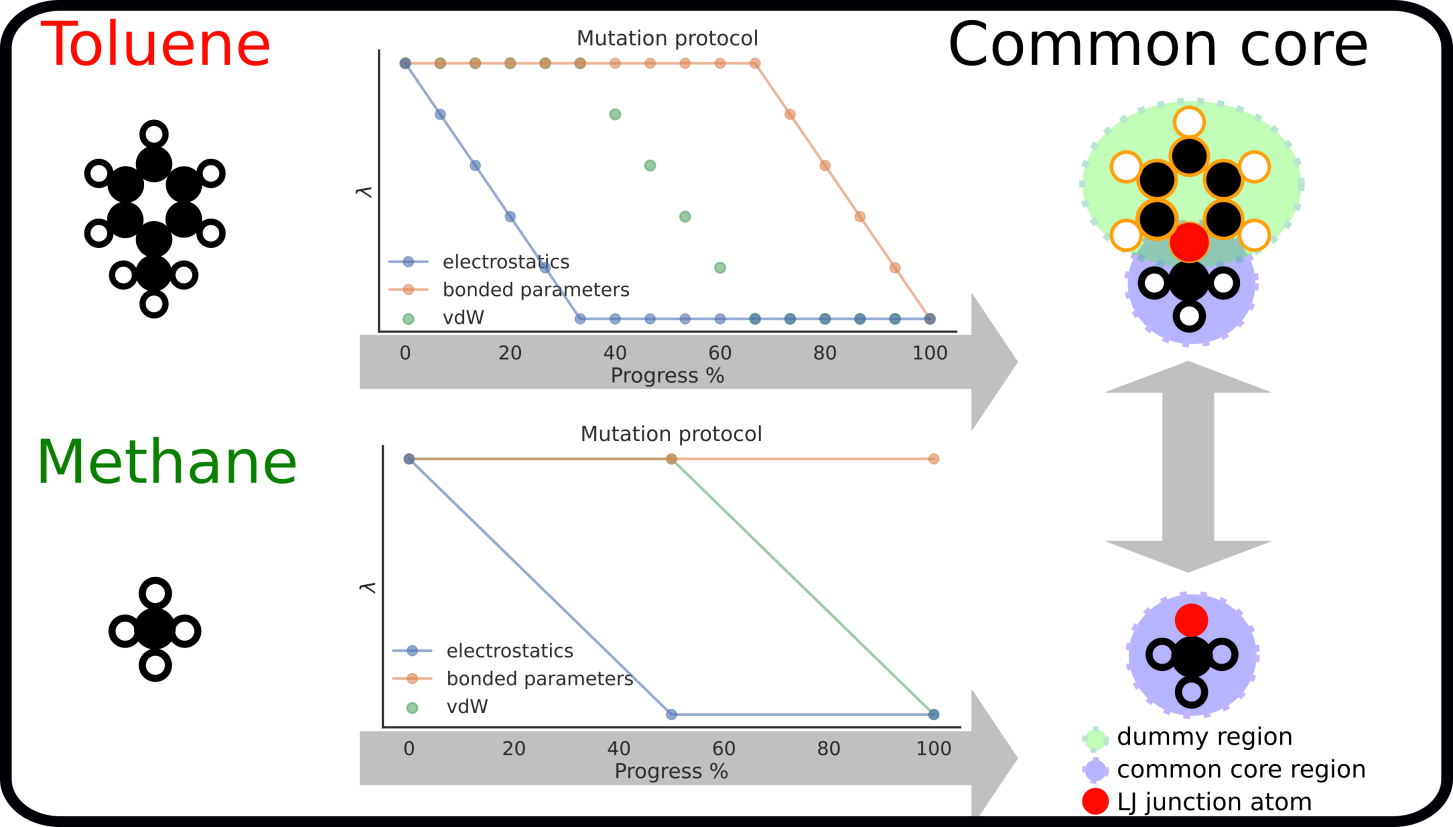
Using the SAI/CC approach the physical end‐states can be formulated without using dummy atoms. The steps needed to compute the relative solvation free energy between toluene and methane using the CC/SAI approach as realized by Transformato are illustrated. Each of the physical molecules is transformed into a common core, shown on the right. The intermediate steps needed in each case are sketched in the plots in the middle: Changes in electrostatics are indicated in light blue, the transformation of LJ interactions to dummy atoms or into the atom type X are indicated in light green, and common core adjustments involving X (here needed only for toluene) are shown in brown. The same intermediates are used in the gas phase and in aqueous solution. The junction atom X is colored in red. The additional dummy atoms present in the common core obtained starting from toluene (top right) have no effect on the relative solvation free energy difference of interest.^20^

Figure 2 illustrates the steps just described for the calculation of the free energy difference between toluene and methane; identical steps were used in the gas phase and in aqueous solution. The target CC, CH${}_{3}$X, is shown at the bottom right of the figure; the junction atom X is colored in red. The steps to transform methane to the CC are shown in the lower half of the figure. The necessary changes in charge are computed in one step (indicated as a single dot in the light blue line), followed by the single change in LJ parameters (H → X, indicated in light green). By contrast, for toluene most of the benzyl ring needs to be transformed into dummy atoms; the ring atom bound to the methyl group becomes the junction atom X. The necessary steps are outlined in the top half of the figure. First, the charges of the benzyl ring are switched off in multiple steps (light blue dots). Then, the LJ interactions of these atoms are turned off in several steps, plus the change C → X is carried out (light green dots). At this point, the bonded parameters involving X are still those of the aromatic carbon. We modify these parameters during additional states (brown dots) into those of a methyl hydrogen interacting with the atoms in the CC. Since the contributions from the dummy atoms present in the CH${}_{3}$X CC obtained as the end‐state of the toluene transformation are identical in the gas phase and aqueous solution, the two CCs shown on the right in Figure 2 are equivalent with respect to the relative solvation free energy difference between toluene and methane, the quantity we want to compute. The detailed number of intermediate states for each of the three stages (turning off electrostatic and vdW interactions, as well as the adjustment of the CC region) and each of the systems is given in Table SI1.

#### 3.2.3 Sampling of alchemical states

Each alchemical state was sampled using Langevin dynamics^37^ at 303.15 K for 2 ns with a 1 fs time step and a friction coefficient of 1/ps; coordinates were saved every 100 steps. Simulations for the solvated system were performed under periodic boundary conditions in a box of TIP3 waters^38^, ^39^ with an initial side length of 30 Å in the isothermal–isobaric ensemble at 1 bar. In calculations with CHARMM we used a Langevin piston barostat,^40^ for OpenMM a Monte Carlo barostat.^41^, ^42^ Waters were kept rigid throughout the simulation utilizing the SHAKE^43^ (CHARMM) or the SETTLE^44^ (OpenMM) algorithm. In line with the protocols used by Loeffler et al.,^15^ the solutes were completely flexible. In the vacuum simulations, no cut‐off was applied to the non‐bonded interactions. In solution, Coulomb interactions were calculated using the particle‐mesh Ewald (PME) method^45^ on a 36 × 36 × 36 grid (CHARMM) and a fractional error tolerance of 0.005 (OpenMM). LJ interactions were switched smoothly to zero between 10 and 12 Å. Calculations with CHARMM were carried out both with the “vswitch”, as well as the “vfswitch” tapering function for the LJ interactions; in the OpenMM calculations we employed “vfswitch”, provided as a custom energy routine from the CHARMM‐GUI server^33^, ^34^ (cf. Overview of simulations). In addition, we used the switching function implemented in OpenMM (“switch”), as well as a hard truncation of LJ interactions (“no‐switch”) at 12 Å. No LJ long‐range corrections were applied to the simulations.

Starting coordinates for the simulations at each intermediate state were obtained as follows. A NVT equilibration simulation of 125 ps length was carried out for the physical end‐state. Before each production simulation of an alchemical state, the coordinates were optimized using the L‐BFGS algorithm in OpenMM or the steepest descent and adopted basis Newton–Raphson minimizer in CHARMM. For each state sampling was carried out with OpenMM and CHARMM, using the LJ switching functions as described. Simulations of each state/condition were repeated five times, using different initial random velocities. A detailed description of the parameters of each system, the mutations, and the input files for each state along the alchemical path can be found in https://github.com/wiederm/Transformato-systems.

#### 3.2.4 Calculating relative free energy differences

Free energy estimates between each of the solutes shown in Figure 1 and the respective CC in the gas phase and in aqueous solution were calculated using the multi‐state Bennett acceptance ratio (MBAR) method as implemented in the pymbar package.^26^ Each alchemical state *λ*_k_ was simulated for 2 ns. In each simulation trajectories containing 20,000 coordinate sets were saved, of which only every third frame of the final 75% were considered for analysis; that is, for each state 5000 frames were processed by pymbar.

For each configuration sample *x*, and each alchemical state *λ*, we computed the reduced potential *u*(*x*,*λ*) to form the *N* × *K* matrix of inputs for MBAR, where *N* is the number of snapshots used and *K* the number of alchemical states *λ*_k_ for a given transformation. Thus, $N=\sum_{k=1}^{K}N_{k}$, with *N*_*k*_ = 5000 snapshots per *λ* state as just described. To implement this efficiently for CHARMM, a single merged trajectory with all configuration samples *x* from each alchemical state *λ* was generated using mdtraj.^46^ This facilitated analysis as for each of the five repetitions for each alchemical transformation only a single trajectory had to be post‐processed for each of the alchemical states.

Solvation free energy differences between two solutes A, B, and their CC were combined according to Equation 3 to obtain ΔΔ*G*(*A* → *B*). The individual values for solute A and B used for Equation 3 were obtained using a thermodynamic cycle (i.e., calculating Δ*G*_vac_ and Δ*G*_solv_ from the physical end‐state to the CC structure as shown in SI Figure SI3). Since calculations were repeated five times the average values for Δ*G*_vac_ and Δ*G*_solv_ for solute A and B were used to calculate ΔΔ*G*(*A* → *B*). The final standard deviation was obtained by Gaussian error propagation.

### 3.3 Absolute solvation free energy calculations

Absolute solvation free energies for each of the compounds were computed with the PERT module of CHARMM. Here the soft‐core potential implemented in PERT was used.^11^ System size, treatment of non‐bonded interactions, thermostat, and barostat settings were analogous to the calculations described above. Similarly, each free energy simulation was repeated five times. A total of 21 alchemical states were used for each calculation. At each state, an equilibration phase with 200 ps was followed by 2 ns production phase, during which ${\left\langle \partial U/\partial \lambda \right\rangle }_{\lambda }$ was evaluated by PERT on the fly. Free energy differences were calculated using thermodynamic integration. The ${\left\langle \partial U/\partial \lambda \right\rangle }_{\lambda }$ values were fitted using natural cubic splines, which were then integrated analytically. Details, including the calculation of error estimates, can be found in Section 4 of SI of Ref. 20.

## 4 RESULTS AND DISCUSSION

**FIGURE 3:**
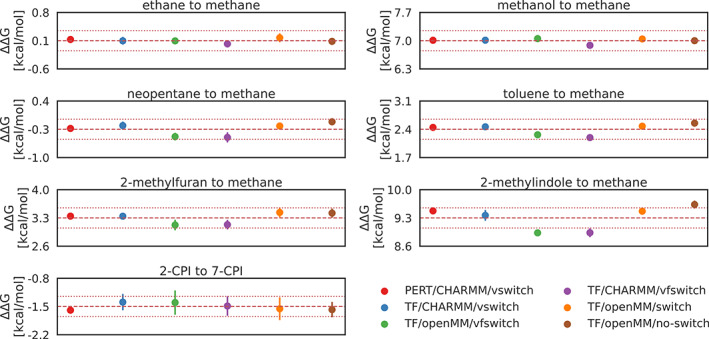
Comparing the ΔΔG values for six different approaches described in the methods section show good agreement on the investigated systems. Each of the free energy calculations was repeated five times and the average of the obtained ΔΔG estimates and its standard deviation is plotted. The dashed red line indicates the total average of the six described approaches and the thin, dotted red lines mark the ±0.25 kcal/mol interval around the average. Results generated with Transformato (abbreviated with TF in the legend) and OpenMM used either the OpenMM native switching function (TF/OpenMM/switch), the implementation of the “vfswitch” function (TF/OpenMM/vfswitch) or no‐switching function (TF/OpenMM/no‐switch), results generated with CHARMM used the “vswitch” (TF/CHARMM/vswitch) or “vfswitch” (TF/CHARMM/vfswitch). In addition to the alchemical path generated using Transformato we also calculated absolute solvation free energies with the PERT module of CHARMM (PERT/CHARMM/vswitch).

All relative solvation free energy differences computed with CHARMM and OpenMM, using various tapering functions for the LJ interactions, as well as reference results are summarized graphically in Figure 3. The raw data from which the plots were generated can be found in Table SI2 of SI. The underlying pymbar framework provides for each individual free energy calculation from physical state to the CC an overlay plot for the alchemical states and an accumulated free energy plot for the transformation; an example is shown in Figure SI1 of SI.

To validate the CC/SAI approach using Transformato we first compared to results obtained with the established PERT module implemented in CHARMM with identical end‐state definition and parameter set. In particular, we compare the relative solvation free energies using the CHARMM back end of Transformato utilizing the “vswitch” switching function (shown in Figure 3 in blue) to the ΔΔG values generated with PERT/CHARMM/vswitch (shown in red) as the difference of the two absolute solvation free energies. The two approaches give ΔΔG estimates for all systems which agree well within statistical error estimates, demonstrating the correctness of the CC/SAI approach as implemented in Transformato.

Next, we tested whether the two MD engines currently supported by Transformato, CHARMM and OpenMM, led to results that do agree within statistical error bars. Here we employed the “vfswitch” LJ switching function available in both MD engines. These results are shown in Figure 3 in purple (TF/CHARMM/vfswitch) and green (TF/OpenMM/vfswitch). Again, the relative solvation free energy differences agree within their standard deviation, with a single exception, the free energy difference between methanol and methane. However, for this transformation the net deviation is very small (0.11 kcal/mol) and the standard deviation extremely low (see Table SI2). For completeness we also report the relative solvation free energy differences obtained using the native switching function in OpenMM (TF/OpenMM/switch) (shown in orange in Figure 3) and with a hard cut‐off (TF/OpenMM/no‐switch) (shown in brown in Figure 3).

Overall, there is surprisingly little variability in the ΔΔG estimates obtained with different switching functions. All relative solvation free energy differences for the pairs ethane/methanol/neopentane/toluene/2‐methylfuran to methane and 2‐CPI to 7‐CPI lie within the ±0.25 kcal/mol interval around the average ΔΔG values (for each system the total average is the average overall values generated with the six different approaches). Only for 2‐methylindole the average values of the five runs reach outside this ±0.25 kcal/mol interval. This agreement may in part be the result of fortunate error compensation arising from the use of thermodynamic cycles, as previously observed; for example, Refs. 47, 48 Differences between results obtained with the various switching methods might be more pronounced for absolute free energies.

While all results for 2‐CPI to 7‐CPI lie within the ±0.25 kcal/mol interval about the average, their standard deviations are relatively high. Since this is the most complex transformation, this is not completely unexpected. However, compared to the analogous mutation from 2‐methylindole to the methane‐like CC, we used fewer steps to turn off vdW interactions (see Table SI1 in SI). When repeating the simulations using vfswitch to truncate LJ interactions with the protocol used for 2‐methylindole (10 instead of 7 steps to turn off vdW interactions), the overall standard deviation was reduced from 0.24 to 0.17 kcal/mol (CHARMM/vfswitch) and from 0.30 to 0.18 kcal/mol (OpenMM/vfswitch, see Table SI2).

Since different force fields were used, we cannot compare our results directly to those of Loeffler et al.^15^ However, it is of interest to take a look at the variability of the free energy estimates obtained with the different software programs used in Ref. 15 and the two supported back ends and different treatments of LJ interactions used in this work. In Figure SI2, we show density plots for the distribution of the relative solvation free energy estimates from Loeffler et al.^15^ and our results reported in Figure 3. The offset in the average values is a direct consequence of the force fields used. The variability of the results, reflected by the widths of the density distributions, on the other hand, is quite similar.

## 5 CONCLUSION

We presented a Python package called Transformato that is able to generate semi‐automatically the alchemical path(s) connecting two or more molecules in a given environment. The results of this work were obtained with an early version of Transformato and serve as the proof of concept of the CC/SAI approach. Transformato is developed as an open source project, see code and data availability below. We validated our methodology and its implementation by using the benchmark set of seven different mutations also used by Loeffler et al.^15^ Within statistical error bars we obtained relative solvation free energies that agreed excellently with results of reference calculations using the PERT module of CHARMM.

Using CC/SAI, that is, Transformato, the end‐states are the true physical molecules without dummy atoms. In traditional single topology setups of alchemical transformations the correct treatment of dummy atom parameters is not trivial. Systematic errors resulting from non‐redundant bonded parameters are a possibility when naively keeping all bonded parameters for the dummy region.^20^ Similarly, the CC/SAI approach avoids the need for hybrid topologies at the end‐states when two chemical moieties are present at the same time, as is typically the case in certain forms of dual topology setups.

The combination of the CC approach with SAI makes it possible to use, in principle, any biomolecular MD program as the back end for Transformato. CHARMM and OpenMM are the most frequently used programs in our groups, and adapting input generated by the CHARMM‐GUI web server^33^, ^34^ is straightforward. At present, Transformato is not tied to CHARMM‐GUI's free energy calculator.^49^ Currently, Transformato writes inputs for the intermediate states in CHARMM format, in particular the PSF and parameter files, though adding the capability to write other formats would be straightforward. Programs, which have support for CHARMM file formats, such as NAMD, could be supported by Transformato easily. The CC/SAI approach in general and Transformato in particular should not be viewed as a front end to dedicated programs to compute free energy differences, but as a tool to carry out FES with almost any MD program. We do neither require nor use any alchemical FES related functionality of the underlying program. Since only minor modifications to input files for supported MD programs, rather than changes at the code level are needed, extending Transformato's functionality to, for example, the calculation of relative binding free energies, both for globular proteins, as well as membrane proteins is straightforward. Work in this direction is currently ongoing.

## Supporting information


**Appendix** **S1**: Supporting Information.

## Data Availability

Python package used in this work (release v0.1): https://github.com/wiederm/transformato. Data and notebooks to reproduce the plots/figures (release v0.1): https://github.com/wiederm/transformato-systems.

## Endnotes

^1^

https://github.com/choderalab/openmmtools.

^2^

https://github.com/choderalab/perses.

^3^

https://academiccharmm.org/documentation/version/c46b1/domdec.
