# Supplementary material for: Alchemical free energy simulations without speed limits. A generic framework to calculate free energy differences independent of the underlying molecular dynamics program
Source: J Comput Chem. 2022 Apr 29;43(17):1151–60. doi: 10.1002/jcc.26877 (PMC9323469; doi:10.1002/jcc.26877)
Supplement: Supplementary file 1 — Appendix S1: Supporting Information. [file JCC-43-1151-s001.pdf]

10 Supplementary Materials

Table S.I.1: Number of intermediate states for each mutation from physical molecule to the respective common core (either methyl-cc or cyclopentyl-cc). The physical starting-state always needs to be added, indicated by (+1) in the rightmost column. Calculations for 2-CPI and 7-CPI to cyclopentyl-cc were performed twice: one set of calculations used 7 vdW mutation steps, the other 11 steps (one for each atom) to turn the indole into a non-interacting group.

**Mutation**	electrostatics	vdW	common core adjustments	Total
methane → methyl-cc	1	1	-	2 (+1)
ethane → methyl-cc	5	2	5	12 (+1)
methanol → methyl-cc	5	2	5	12 (+1)
neopentane → methyl-cc	5	5	5	15 (+1)
toluene → methyl-cc	5	5	5	15 (+1)
2-methylfuran → methyl-cc	5	6	5	16 (+1)
2-methylindole → methyl-cc	5	10	5	20 (+1)
2-cpi → cyclopentyl-cc	5	7	5	17 (+1)
7-cpi → cyclopentyl-cc	5	7	-	12 (+1)
2-cpi → cyclopentyl-cc	5	10	5	20 (+1)
7-cpi → cyclopentyl-cc	5	10	-	15 (+1)

For Peer Review

Table S.I.2: Detailed summary of relative solvation free energy differences depicted graphically in Fig. 3 of the main manuscript. $\Delta\Delta G$ values were calculated using two independent approaches: as the difference of two absolute solvation free energy calculations using the `PERT` module of `CHARMM`, and relative solvation free energies using `Transformato` with `OpenMM` and `CHARMM` as the MD engine. To calculate the free energy with `PERT` thermodynamic integration was used, the free energy values for `Transformato` were generated using the multi-state Bennett acceptance ratio (MBAR) method as implemented in the `pymbar` package.[26] All calculations were repeated five times and the average free energy difference its and standard deviation is reported.

	`PERT`	TF/`CHARMM`		TF/`OpenMM`		
	vswitch	vswitch	vfswitch	no-switch	switch	vfswitch
$\Delta\Delta G$(ethane, methane)	0.10±0.03	0.07±0.10	-0.01±0.09	0.05±0.07	0.14±0.11	0.07±0.08
$\Delta\Delta G$(methanol, methane)	7.05±0.03	7.05±0.05	6.92±0.05	7.04±0.06	7.08±0.07	7.09±0.09
$\Delta\Delta G$(neopentane, methane)	-0.23±0.03	-0.16±0.10	-0.45±0.13	-0.06±0.09	-0.17±0.08	-0.43±0.09
$\Delta\Delta G$(toluene, methane)	2.40±0.04	2.41±0.04	2.15±0.09	2.51±0.05	2.43±0.08	2.19±0.05
$\Delta\Delta G$(2-methylfuran, methane)	3.31±0.05	3.31±0.08	3.1 ±0.12	3.39±0.11	3.40±0.12	3.09±0.13
$\Delta\Delta G$(2-methylindole, methane)	9.47±0.05	9.36±0.13	8.93±0.11	9.63±0.10	9.46±0.09	8.93±0.07
$\Delta\Delta G$(2-cpi, 7-cpi)[a]	-1.62±0.11	-1.42±0.20	-1.52±0.24	-1.61±0.19	-1.58±0.28	-1.43±0.30
$\Delta\Delta G$(2-cpi, 7-cpi)[b]			1.52±0.17			-1.34±0.18

[a]) Results for 2-CPI to 7-CPI as reported in Fig. 3, turning off the vdW interactions of the indole ring in 7 steps.
[b]) Calculations using the vfswitch method to truncate Lennard-Jones interactions were repeated with a more elaborate protocol; 10 steps to turn off vdW interactions. See Table S.I.1 for additional details.

For Peer Review

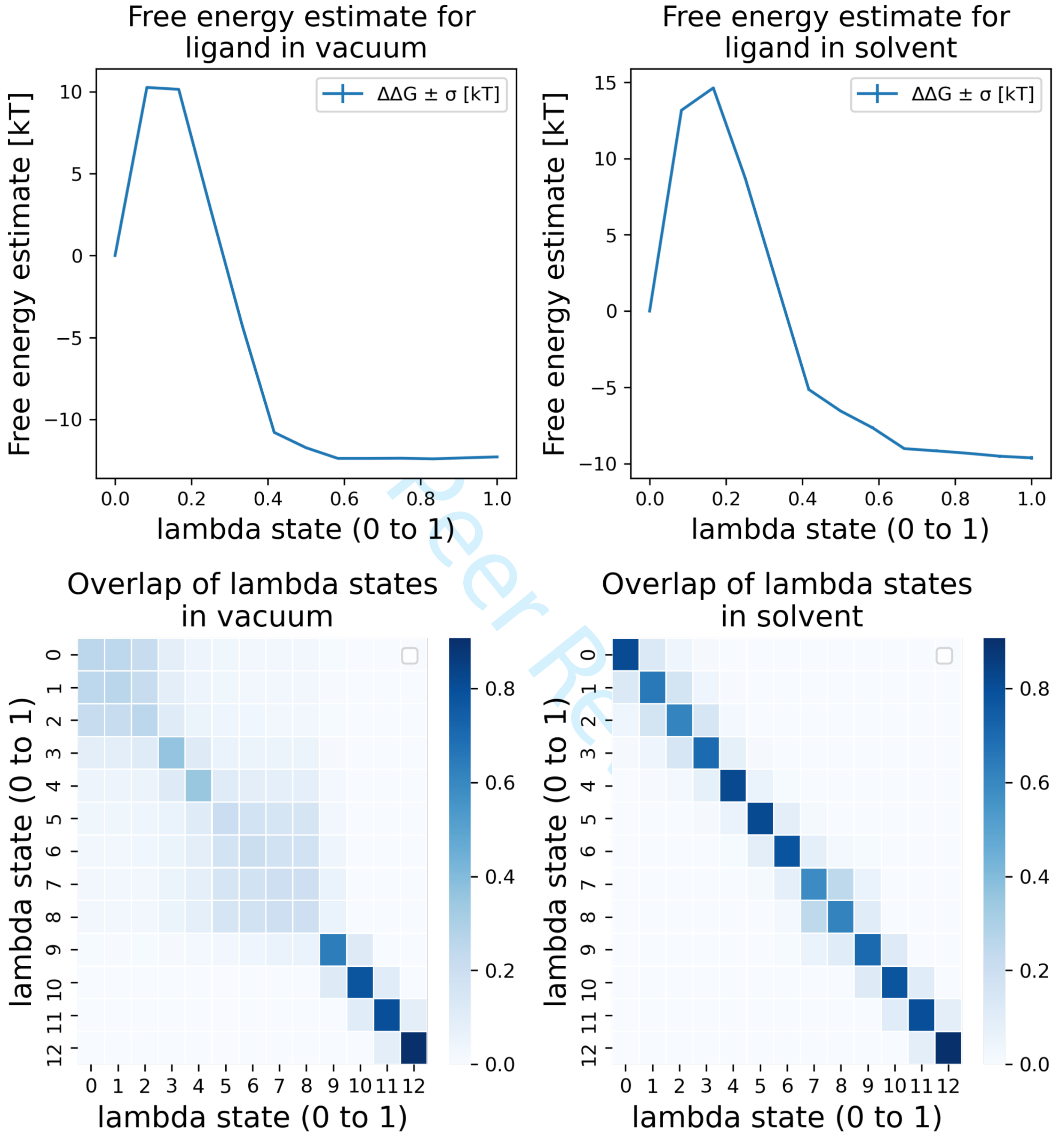

Figure S.I.1: Results returned from an relative alchemical free energy calculation transforming toluene to methane-X . The top panel shows the accumulated free energy estimate and the free energy estimate uncertainty obtained from the MBAR estimate for the vacuum and solvent phase. The bottom panel shows the overlap plot along the alchemical path for both phases.

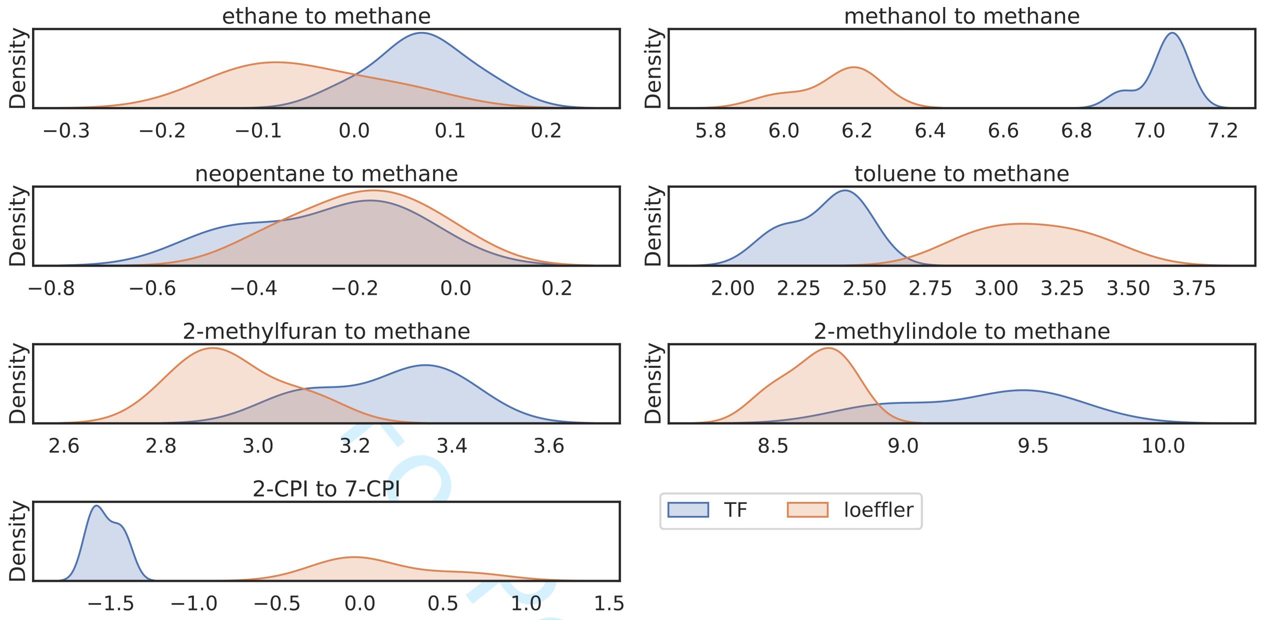

Figure S.I.2: Density plot of the relative solvation free energy estimates reported in Loeffler et al.[15] and used in Figure 3. The distributions are offset because different force field parameters were used for the two set of calculations.

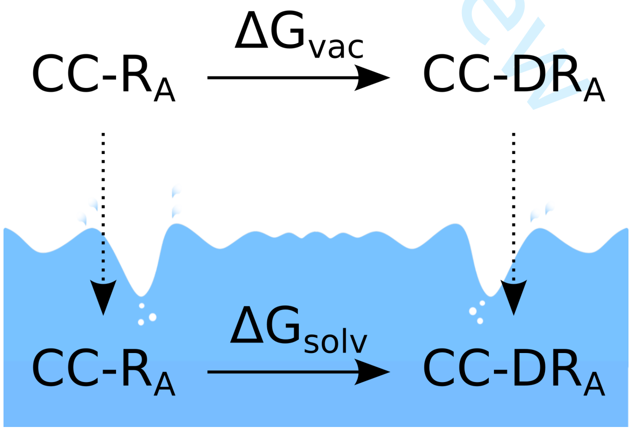

Figure S.I.3: Thermodynamic cycle used to calculate the free energy of a compound ($CC - R_A$) to its common core structure ($CC - DR_A$). To obtain ΔΔG(CC-R_A → CC-DR_A) as used in Eq. 3 we calculate $\Delta G_{solv} - \Delta G_{vac}$.
